# High-Performance UV-Vis Light Induces Radical Photopolymerization Using Novel 2-Aminobenzothiazole-Based Photosensitizers

**DOI:** 10.3390/ma14247814

**Published:** 2021-12-17

**Authors:** Alicja Balcerak, Janina Kabatc, Zbigniew Czech, Małgorzata Nowak, Karolina Mozelewska

**Affiliations:** 1 Department of Organic Chemistry, Faculty of Chemical Technology and Engineering, Bydgoszcz University of Science and Technology, Seminaryjna 3, 85-326 Bydgoszcz, Poland; nina@pbs.edu.pl; 2 International Laboratory of Adhesives and Self-Adhesive Materials, Department of Chemical Organic Technology and Polymeric Materials, Faculty of Chemical Technology and Engineering, West Pomeranian University of Technology in Szczecin, Pułaskiego 10, 70-322 Szczecin, Poland; nowak.malgorzata@zut.edu.pl (M.N.); karolina_mozelewska@zut.edu.pl (K.M.)

**Keywords:** UV-Vis light photoinitiators, 2-aminobenzothiazole derivatives, squaraine dyes, iodonium salts, radical polymerization

## Abstract

The popularity of using the photopolymerization reactions in various areas of science and technique is constantly gaining importance. Light-induced photopolymerization is the basic process for the production of various polymeric materials. The key role in the polymerization reaction is the photoinitiator. The huge demand for radical and cationic initiators results from the dynamic development of the medical sector, and the optoelectronic, paints, coatings, varnishes and adhesives industries. For this reason, we dealt with the subject of designing new, highly-efficient radical photoinitiators. This paper describes novel photoinitiating systems operating in UV-Vis light for radical polymerization of acrylates. The proposed photoinitiators are composed of squaraine (SQ) as a light absorber and various diphenyliodonium (Iod) salts as co-initiators. The kinetic parameters of radical polymerization of trimethylolpropane triacrylate (TMPTA), such as the degree of double bonds conversion (C_%_), the rate of photopolymerization (R_p_), as well as the photoinitiation index (I_p_) were calculated. It was found that 2-aminobenzothiazole derivatives in the presence of iodonium salts effectively initiated the polymerization of TMPTA. The rates of polymerization were at about 2 × 10^−2^ s^−1^ and the degree of conversion of acrylate groups from 10% to 36% were observed. The values of the photoinitiating indexes for the most optimal initiator concentration, i.e., 5 × 10^−3^ M were in the range from 1 × 10^−3^ s^−2^ even to above 9 × 10^−3^ s^−2^. The photoinitiating efficiency of new radical initiators depends on the concentration and chemical structure of used photoinitiator. The role of squaraine-based photoinitiating systems as effective dyeing photoinitiators for radical polymerization is highlighted in this article.

## 1. Introduction

Currently, a large number of polymer materials is produced by photopolymerization [1]. Generally, photochemically induced polymerization is defined as a process in which reactive species (radicals or ions) formed from light-activated molecules, called photoinitiators (PI), initiate a series of chemical reactions and as a result transform liquid monomers into cross-linked polymer structures.

The photopolymerization is considered as one of the most widespread, modern and rapidly developing technologies used in the chemical industry [2,3,4]. Photoinitiated polymerization reactions show a huge potential in the simple and fast synthesis of polymeric materials with specified features. In comparison with the conventional curing techniques, the interest towards photochemically initiated polymerization is constantly gaining importance [5]. The increasing popularity of photopolymerization is related to numerous unique advantages, such as low economic costs [6,7], high efficiency [8,9], the possibility of spatial and temporal control over the process [10,11] and a fast-curing rate [12]. These techniques are referred to as eco-friendly because of the possibility of using solvent-free polymerizable compositions with zero or a very low index of volatile organic compounds (VOCs), which are one of the major sources of environmental pollution. Moreover, there is the possibility of the easy recycling of waste generated during the production of process [13,14].

**Figure 1:**
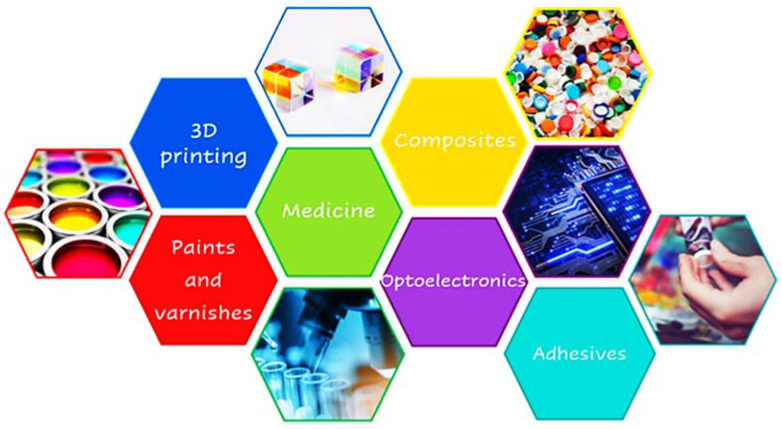
Areas of applications of photopolymerization processes.

Due to many advantages, the light-activated polymerization has a wide range of applications. This process is used in many areas of science and technology, such as: medicine, paint and varnish industry, printing, production of adhesives, nanotechnology, electronic sector and many others [15,16]. Some examples of applications of photopolymerization in different areas are presented in Figure 1.

One of the main uses of the photopolymerization process in medicine is the production of dental fillings. Novel, durable and aesthetic light-cured dental composites displaced the traditional amalgams and became the basic materials used to fill cavities in teeth. However, the requirements for new composites are still enormous and pose huge challenges for designing dental fillings [17,18]. The light-cured dental fillings should show high durability, attrition strength, as well as chemical and physical resistance factors, such as: salivary enzymes, acids and bacterial metabolism products and others. Moreover, it is extremely important that these types of materials exhibit high biocompatibility in relation to oral tissues, good dimensional stability during crosslinking and resistance for yellowing [19]. 

The subject of design and the development of new hydrogels is also more and more popular. These elastic and highly hydrated biomaterials possess great and unique properties, which enables them usage in regenerative medicine [20]. Especially important features of hydrogels are: biocompatibility, biodegradability, extreme water-binding capacity and the ability to adjust the physicochemical properties. Hydrogels exhibit an excellent ability to heal wounds, which was confirmed by a large group of scientists [21,22,23].

The photopolymerization is a key process in the production of various types of polymer coatings. Innovative varnishes with excellent antibacterial and antimicrobial properties for dentistry [24,25], novel paints for the degradation of organic pollutants in water [26], biocompatible film-forming polymer adhesives activated by natural sunlight [27], and programmable shape-shifting 3D structures [28] are just a few examples of new inventions that represent an important step towards the progress of new technologies. 

Notably, in recent years, a dynamic progress of 3D printing technique was observed, which offers many benefits compared to traditional manufacturing processes of novel polymer materials [29,30]. First of all, the use of 3D printing techniques enables us to accelerate the time of introducing novel products to the worldwide market. Moreover, this technology eliminates the need of expensive apparatus and thus significantly decreases the production costs. The additional benefits include: accuracy, speed and flexibility towards manufacturing a wide range of materials. What is important is that 3D printing guarantees less waste production than traditional methods [31,32]. An interesting work about the application of these technique was presented in 2021 by Bai and co-workers [33]. A group of scientists described 3D concrete printing (3DCP), which is a new and promising construction technology. The researcher group introduced the possibility of using underutilized solids and waste solid aggregates for the production of concrete. Three-dimensional printing has become extremely important also during the current COVID-19 pandemic, what is confirmed by numerous publications on this topic [34,35,36,37]. The use of this technology has proved invaluable in eliminating the shortage of basic personal protective supplies and healthcare equipment for medical personnel. The 3D printing technique is used to produce medical face shields, respirators, biodegradable mask filters and 3D bioprinted tissue models for coronavirus research. The dynamics of the research in the field of 3D printing has become a very important aspect in the fight against the pandemic.

Numerous examples of the use of photopolymerization processes in various areas of science and technology show how important these topics are and prove the high potential of this technology in the production of various polymer materials. Hence, searching for new photoinitiating systems (PISs) and designing new photocurable compositions is a key aspect for progress in the development of novel techniques such as photopolymerization.

Typical photocurable composition is comprised of monomer or mixtures of monomers and oligomers, photoinitiator and other ingredients: solvents, fillers, substances improving the stability of formulations and others [38,39]. Nowadays, a wide variety of monomers is commercially available. The most commonly used compounds are acrylates and methacrylates, epoxides, esters, urethanes, etc. A large number of photoinitiators for radical as well as ionic polymerization have been already described in the literature. However, a large number of scientific works is directed towards searching for new, high-performance photoinitiating systems. Next generation photoinitiators exhibit high activity even under low intensities of light. Moreover, an increasing number of novel compounds work not only in the range of ultraviolet (UV), but also in visible light (Vis) [40,41,42]. 

The wide group of photoinitiators are systems based on dyes acting as light absorbers. The introduction of dye molecules into the photoinitiating system enables a shift in the band of absorption towards longer wavelengths and, significantly, expands the area of application of the photoinitiator. The dye molecule acts as a sensitizer for other compounds, such as co-initiators, which work as electron donors or electron acceptors. In this type of system, the main process leading to the generation of initiating radicals is an electron transfer process (ET) [3]. 

**Figure 2:**
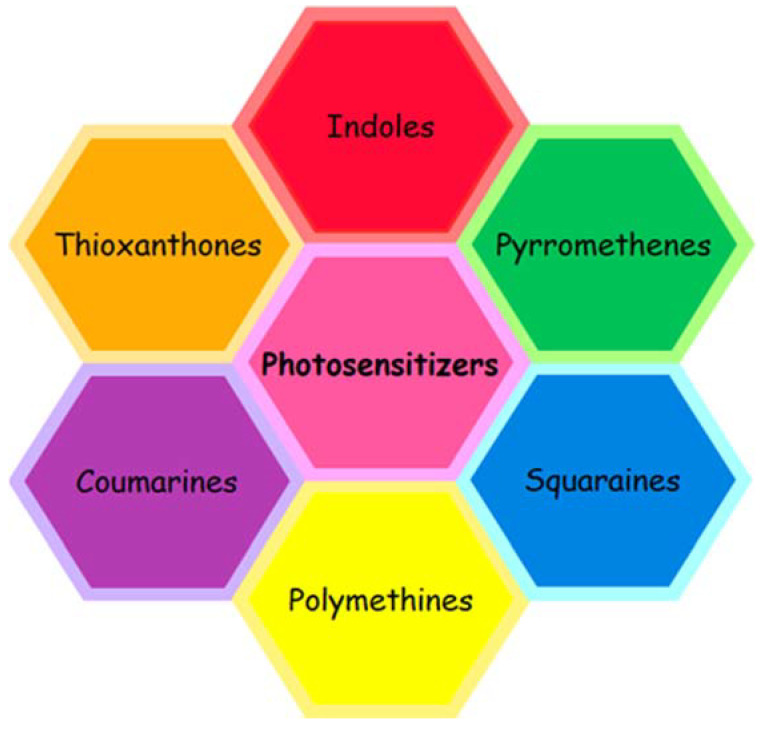
Examples of dyes used as photosensitizers in dyeing photoinitiating systems [43].

The example of highly efficient initiators dedicated to radical polymerization are the two-component PISs containing of sensitizer molecule and co-initiator. In such bimolecular systems, the role of light absorbers can be played by various types of organic dyes, which was presented in Figure 2 [43].

The application of many of these compounds as photosensitizers in dye-based photoinitiating systems was described in the literature. However, the information about photosenzitizers in the form of squaraines (SQs) are limited. The first paper mentioning the use of squaraine dyes as photosensitizers for radical polymerization was reported in 2004 by Wang and co-workers [44]. In 2021, Giacoletto and co-workers [45] presented an overview paper regarding recent advances on squaraine-based photoinitiators. It turns out that squaraines are a promising group of useful dyes for various innovative applications because of their unique features, such as the easiness of the methods of synthesis, the low costs of manufacturing and good photochemical stability [45,46]. For these reasons, the design of novel squaraine acid derivatives seems to be really important. 

The 2-aminobenzothiazole derivatives are an interesting group of compounds showing a wide range of applications due to their specific properties. For example, in 2016 Joseph and Boomadevi Janaki [47] described new copper complexes of Schiff base ligands of 2-aminobenzothiazole derivatives. These compounds were synthesized by the condensation of Knoevenagel of acetoacetanilide and 2-aminobenzothiazole. The cooper complexes are characterized by a wide range of absorption, from 200 nm to ca. 800 nm. Moreover, all synthesized compounds show high antioxidant activity, antibacterial and antifungal properties. For this reason, it is possible to use of these cooper complexes based on 2-aminobenzothiazole to decrease ROS levels or reduce oxidative stress in Alzheimer’s patients [47]. 

In this paper, we focused on determining the efficiency of novel two-component systems based on newly synthesized 2-aminobenzothiazole derivatives for the photoinitiation of radical polymerization of acrylate monomer. We decided to use these dyes as UV-Vis light absorbers and combined them with various iodonium salts to obtain a high performance photoinitiators. The similar compounds were used as sensitizers for radical polymerization of 1,6-hexanediacrylate (HDDA) and gave promising results of kinetics of photopolymerization reaction, which was described by Zhao and co-workers in 2020 [48]. Novel *S*-benzoheterocycle thiobenzoates photoinitiators showed an excellent photoinitiating ability and cured polymeric films possessed comparable or better mechanical properties in comparison with commercially available photoinitiators, such as benzophenone (BP) and irgacure 184 [48]. Due to the high-performance of photoinitiating systems composed of dye as sensitizer and co-initiator in the form of iodonium salt, what was proposed by Zhang and co-workers in 2019 [49] we examined the kinetics of the radical polymerization of trimethylolpropane triacrylate (TMPTA) using novel photoinitiators based on 2-aminobenzothiazole derivatives and various diphenyliodonium salts. 

## 2. Materials and Methods

### 2.1. Materials

**Scheme 1:**
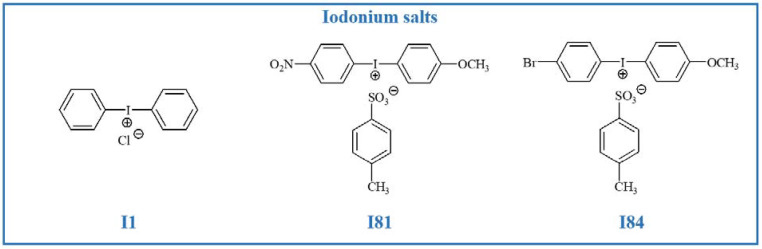
The chemical structures of co-initiators.

All reagents used for the synthesis of 2-aminobenzothiazole derivatives, such as: 3,4-dihydroxy-3-cyclobutene-1,2-dione (squaric acid), 2-aminobenzothiazole, 2-amino-6-bromobenzothiazole, 2-amino-6-methylbenzothiazole, 1-butanol and toluene were purchased from Aldrich Chemical Co. (Milwaukee, WI, USA) and used without further purification. The spectroscopic grade solvents *N,N*-dimethylformamide (DMF), and 1-methyl-2-pyrrolidinone (MP) were supplied by Alfa Aesar (Heysham, Lancashire, UK) and Aldrich Chemical Co. (Milwaukee, WI, USA). Diphenyliodonium chloride (I1) was purchased from Acros Organics (Carlsbad, CA, USA) and other iodonium salts, such as: (4-methoxyphenyl)-(4-nitrophenyl) iodonium *p*-toluenesulfonate (I81) and (3-bromophenyl)-(4-methoxyphenyl)iodonium *p*-toluenesulfonate (I84) were synthesized by scientists from Cracow University of Technology, as described in the literature [50]. The chemical structures of iodonium salts I1, I81 and I84 used as co-initiators in photopolymerization experiments are shown in Scheme 1. Trimethylolpropane triacrylate (TMPTA, from Sigma Aldrich, Burlington, MA, USA) was applied as a model acrylate monomer for the composition polymerized by a radical mechanism.

### 2.2. General Procedure for the Synthesis of 2-Aminobenzothiazole Derivatives

**Scheme 2:**
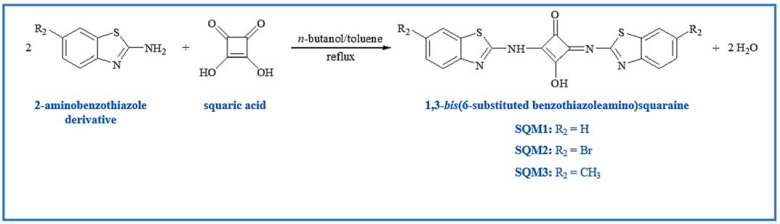
Method of synthesis of 2-aminobenzothiazole derivatives.

The series of novel 2-aminobenzothiazoles, which played a role of photosensitizers in two-component photoinitiating systems, i.e.,: 1,3-*bis*(benzothiazoleamino)squaraine (SQM1), 1,3-*bis*(6-bromobenzothiazoleamino)squaraine (SQM2) and 1,3-*bis*(6-methylbenzothiazoleamino)squaraine (SQM3), was synthesized in one-step reaction. The condensation reaction of 1 eqv. of squaric acid with 2 eqv. of 6-substituted 2-aminobenzothiazoles leads to squaraine dye formation, as presented in Scheme 2.

**Scheme 3:**
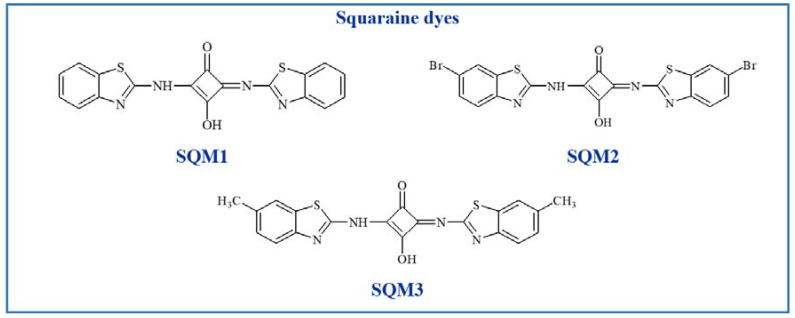
The chemical structures of photosensitizers.

The squaraines SQM1-SQM3 were synthesized, as follows: 1,2-dihydroxycyclobuten-3,4-dione (2.5 mmol) was heated under reflux in a mixture of 1-butanol (40 mL) and toluene (20 mL). The water was distilled off azeotropically using a Dean-Stark trap. After 1 h of heating, an appropriate 6-substituted 2-aminobenzothiazole derivative (5 mmol) was added and the reaction mixture refluxed for additional 4 h. After that, the reaction mixture was cooled and the solvent removed under vacuum. The solid was dried at room temperature [46]. The 2-aminobenzothiazole derivatives were used as photosensitizers in photopolymerization experiments. The chemical structures of these compounds are depicted in Scheme 3. The ^1^H and ^13^C NMR spectra of SQM1-SQM3 compounds are available in Supplementary Materials (Figures S1–S6).

### 2.3. Methods

The chemical structure of synthesized compounds was confirmed by nuclear magnetic resonance (NMR) technique. The ^1^H and ^13^C NMR spectra were registered using an Ascend III spectrometer operating at 400 MHz (Bruker, Billerica, MA, USA). Dimethylsulfoxide (DMSO-*d_6_*) was used as solvent and tetramethylsilane (TMS) as internal standard. Chemical shifts (δ) are reported in ppm relative to TMS and coupling constants (*J*) in Hz.

The melting point (uncorrected) was measured on the Böethius apparatus, PGH Rundfunk (Fernsehen Niederdorf KR, Stollberg/Erzgebirge, Sachsen, Germany).

The absorption and fluorescence spectra of squaraine dyes were registered at room temperature in a quartz cuvette (1 cm) using an UV-Vis Cary 60 spectrophotometer (Agilent Technology, Santa Clara, CA, USA) and F-7000 spectrofluorometer (Hitachi, Tokyo, Japan), respectively.

The fluorescence quantum yields of squaraines were determined, as follows: the fluorescence spectrum of diluted solution of dye (A_366 nm_ ≈ 0.1) was registered by excitation at the maximum of the absorption band of the reference. The standard was chosen based on the similarity of the maximum absorption of dye. The fluorescence quantum yields of dye (Φ_dye_) were determined compared with the fluorescence of cumarine 1 in ethanol (λ_ex_ = 366 nm, Φ_ref_ = 0.64 [51]). This parameter was calculated on the basis of Equation (1):

(1) $$\Phi_{\mathrm{fl}}=\frac{\Phi_{\mathrm{ref}}A_{\mathrm{ref}}I_{\mathrm{dye}}n_{\mathrm{dye}}^{2}}{A_{\mathrm{dye}}I_{\mathrm{ref}}n_{\mathrm{ref}}^{2}}$$

where: Φ_ref_ is the fluorescence quantum yield of reference, A_dye_ and A_ref_ are the absorbances of dye and reference at the excitation wavelength, I_dye_ and I_ref_ are the integrated emission intensities for the dye and reference, n_dye_ and n_ref_ are the refractive indexes of the solvents used for the dye and reference, respectively.

The steady-state photolysis experiments were carried out to investigate the interactions between photosensitizer and co-initiator. The changes of the absorption of the photosensitizer and photosensitizer in the presence of an appropriate co-initiator in *N,N*-dimethylformamide (DMF) as a solvent after irradiation with an argon-ion laser at an intensity of 50 mW × cm^−2^ were registered. The absorption measurements after 0 s, 10 s, 30 s, 60 s, 10 min, 20 min, 30 min and 60 min of irradiation were registered using an UV-Vis Cary 60 spectrophotometer (Agilent Technology). The concentration of co-initiator was 1 × 10^−3^ M.

### 2.4. Photopolymerization Experiments

The general procedure for photopolymerization experiments included several fundamental steps:

(a) The synthesis and spectral characterization of novel photosensitizers;
(b) The preparation of polymerizable compositions containing an appropriate concentration of photoinitiator;
(c) The selection of curing conditions of the polymerizing mixture, i.e.,: light source, intensity and range of light radiation and flow of inert gas (nitrogen).

**Figure 3:**
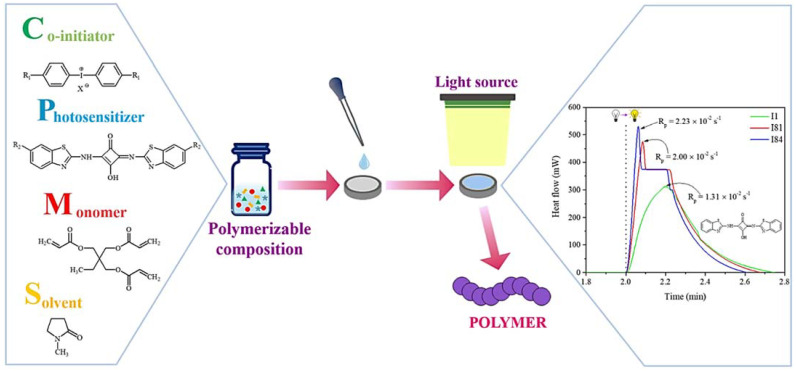
Schematic steps representation of photopolymerization experiments.

The basic steps for the photopolymerization experiments are shown in Figure 3. The preparation of the appropriate polymerizable composition required the synthesis of new photosensitizers. These compounds were prepared according to the general procedure described in Section 2.2. In the next step, the squaraine dyes were combined with different co-initiators. These combinations were two-component photoinitiating systems for radical polymerization of trimethylolpropane triacrylate (TMPTA). The polymerization mixture was composed of 1.8 mL of monomer (TMPTA), 0.2 mL of solvent (MP), appropriate photosensitizer (SQM1/SQM2/SQM3) and co-initiator (I1/I81/I84). The use of 1-methyl-2-pyrrolidinone (MP) was necessary due to poor solubility of dye in monomer. The experiments were carried out for various concentration of photoinitiators: 5 × 10^−4^ M, 1 × 10^−3^ M, 2 × 10^−3^ M and 5 × 10^−3^ M. The monomer composition without a co-initiator was used as a reference sample. 

In order to determine the photoinitiation efficiency of the proposed systems, a regular photo-DSC setup was used. The photopolymerization experiments were carried out using differential scanning calorimeter—DSC Q2000 (TA Instruments, New Castle, DE, USA) connected with TA Q PCA photo unit equipped with a high-presure mercury lamp-OmniCure S2000 (Excelitas Technologies, Waltham, MA, USA). The radiation in the UV-Vis range (300–500 nm) at constant light intensity equal to 30 mW × cm^−2^ was used as a light source. All measurements were performed under isothermal conditions at 25 °C and nitrogen flow of 50 mL × min^−1^. The tested and reference samples weighing 30 ± 0.1 mg were placed into an open aluminum DSC pan and then maintained at the prescribed temperature, i.e.,: 25 °C for 2 min before each measurement run began. The heat evolved during the exothermal reaction and was registered at sampling intervals of 0.05 s per point.

On the basis of the obtained data, the kinetic parameters of photopolymerization process, such as: the degree of monomer conversion (C_%_), the rate of polymerization (R_p_) and photoinitiation index (I_p_) were calculated. The value of C_%_ parameter is directly proportional to the number of reactive moieties in the monomer molecule, which corresponds to the acrylate groups. The conversion percentages were determined on the basis of the integrated area under exothermic peak using Equation (2):

(2) $$C_{\%}=\frac{\Delta H_{t}}{\Delta H_{0}}\times 100$$

where ΔH_t_ is the heat evolved at time t during reaction, ΔH_0_ is the theoretical enthalpy for the complete degree of monomer conversion (for acrylates: ΔH_0_ = 78.0 kJ × mol^−1^ [52]).

The rate of polymerization (R_p_) corresponds to the amount of heat released during the chain reaction. This parameter was estimated using Equation (3):

(3) $$R_{p}=\frac{\mathrm{dH}/\mathrm{dt}}{\Delta H_{0}}$$

where dH/dt denotes how the heat flow evolved during the polymerization reaction.

Taking into account the maximum rate of polymerization (R_p (max)_) and the time required for the maximum rate of heat released in the polymerization reaction (t_max_), the overall ability to the initiation of polymerization reaction (I_p_) may be calculated on the basis of the formula presented below (Equation (4)):

(4) $$I_{p}=\frac{R_{p\;\left(\max\right)}}{t_{\left(\max\right)}}$$

The kinetic parameters of polymerization process expressed by Equations (2)–(4) were used as a determinants for the evaluation of the photoinitiation efficiency of new squaraine-based photoinitiators. On the basis of these parameters, the most effective photoinitiating systems for radical polymerization of acrylate monomers has been detailed in this article.

## 3. Results and Discussion

### 3.1. Characteristics of Photoinitiators

As mentioned above, in this article we proposed new bimolecular photoinitiators composed of squaraine dye as a photosensitizer and iodonium salt in the role of a co-inititiator. The chemical structure of synthesized photosensitizers was confirmed by the NMR technique. The ^1^H and ^13^C NMR spectra clearly confirmed the chemical structure of dyes (SQM1-SQM3). The structure analysis of squaraines is presented below. The ^1^H and ^13^C NMR spectra of all synthesized compounds are available in the ESI file. The structure characteristics data of 2-aminobenzothiazole derivatives are as follows:

▪ 1,3-Bis(benzothiazoleamino)squaraine (SQM1)

^1^H NMR (DMSO-*d_6_*), δ (ppm): 8.49 (s, 1H, -OH); 7.98–7.96 (d, 2H, Ar, *J* = 8 Hz); 7.78–7.72 (m, 2H, Ar); 7.48–7.40 (m, 3H, Ar); 7.34–7.30 (m, 2H, Ar).

^13^C NMR (DMSO-*d_6_*), δ (ppm): 187.2; 171.8; 168.1; 162.8; 146.9; 130.6; 127.4; 126.8; 124.5; 123.0; 122.8; 122.3; 118.9; 116.7; 60.8; 19.1; 18.6; 14.3.

The SQM1 dye was obtained as orange solid, yield: 49.54%, mp. 190–227 °C.

▪ 1,3-Bis(6-bromobenzothiazoleamino)squaraine (SQM2)

^1^H NMR (DMSO-*d_6_*), δ (ppm): 8.21 (s, 1H, Ar); 8.20 (s, 2H, -NH, -OH); 8.04 (s, 1H, Ar); 7.95–7.94 (d, 1H, Ar, *J* = 4 Hz); 7.57–7.54 (dd, 1H, Ar, *J* = 12 Hz); 7.41–7.38 (dd, 1H, Ar, *J* = 12 Hz); 7.29–7.27 (d, 1H, Ar, *J* = 8 Hz).

^13^C NMR (DMSO-*d_6_*), δ (ppm): 187.1; 172.0; 168.0; 162.7; 150.1; 148.1; 133.8; 132.4; 130.0; 129.1; 125.1; 124.1; 121.2; 119.0; 115.8; 113.2.

The SQM2 dye was obtained as orange solid, yield: 80.39%, mp. 206–230 °C.

▪ 1,3-Bis(6-methylbenzothiazoleamino)squaraine (SQM3)

^1^H NMR (DMSO-*d_6_*), δ (ppm): 8.26 (s, 2H, -NH, -OH); 7.79 (s, 1H, Ar); 7.65–7.62 (dd, 1H, Ar, *J* = 12); 7.61–7.60 (s, 1H, Ar); 7.37–7.35 (d, 1H, Ar, *J* = 8 Hz); 7.33–7.29 (m, 1H, Ar); 7.19–7.16 (dd, 1H, Ar, *J* = 12); 2.45 (s, 3H, -CH_3_); 2.39 (s, 3H, -CH_3_).

^13^C NMR (DMSO-*d_6_*), δ (ppm): 187.5; 172.6; 167.3; 161.9; 146.1; 145.3; 133.8; 131.8; 131.0; 128.9; 128.4; 127.6; 122.5; 122.0; 118.6; 116.6; 70.8; 21.4; 21.2; 18.7; 14.0.

The SQM3 dye was obtained as orange solid, yield: 80.42%, mp. 128–153 °C.

One of the most important preconditions of photopolymerization experiments is the selection of a proper light source. For this reason, the spectroscopic properties of photoinitiators were studied.

**Figure 4:**
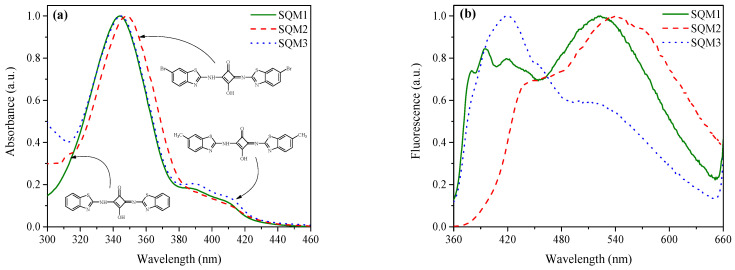
Normalized absorption (**a**) and fluorescence (**b**) spectra of squaraines SQM1-SQM3 in 1-methyl-2-pyrrolidinone (MP) recorded at room temperature.

From the data presented in Figure 4a and summarized in Table 1, it can be concluded that all 2-aminobenzothiazole derivatives absorb in narrow range of spectrum. The absorption bands are intensive and range from 300 nm to ca. 460 nm. The maximum absorption (λ_ab (max)_) of all squaraines is about 345 nm. Moreover, the molar extinction coefficients for SQs achieve high values and are in the order of 2 × 10^4^ dm^3^ × mol^−1^ × cm^−1^ for SQM1 and about 1 × 10^3^ dm^3^ × mol^−1^ × cm^−1^ for other. The spectral data of investigated dyes are summarized in Table 1. The 1-methyl-2-pyrrolidinone was used as solvent in spectroscopic studies.

As shown in Figure 4b, the fluorescence bands are broad and ranging from 360 nm to 660 nm. The position of the fluorescence bands depends both on the structure of the dye, as well as, the type of solvent used. In comparison to the absorption maxima, the values of λ_fl(max)_ for SQM1-SQM3 compounds are different. Interestingly, the squaraine dyes have two characteristic fluorescence maxima. It may be explained by the presence of three emission bands, i.e., free dye, dye-solvent complex and twisted excited state resulting from C-N bond rotation [53]. The main maximum of fluorescence is 523 nm for SQM1, 538 nm for SQM2 and 418 nm for SQM3, respectively.

The Stokes shifts (Δν_St_) reached high values, from 5 × 10^3^ cm^−1^ to 10 × 10^3^ cm^−1^. The fluorescence quantum yields (Φ_fl_) are similar for all studied dyes. As is clearly seen, the proposed squaraines show excellent spectroscopic properties for their application as photosensitizers in photoinitiating systems.

On the other hand, the iodonium salts: I1, I81 and I84 absorb light below 300 nm [3]. Due to the absorption range of these compounds in the ultraviolet, usually the iodonium salts need to be combined with other molecules, which absorb light in the visible range of spectrum. Therefore, the introduction of squaraine dye into photoinitiating system is necessary, because it shifts the sensitivity of the photoinitiator towards longer wavelengths. The light source emitted from the high-pressure mercury lamp (OmniCure S2000) cover the range from 300 nm to 600 nm and in this case overlaps with the absorption region of squaraine dye. To summarize, the combination of squaraine dye with iodonium salt is a promising system, which can be used for initiation of the radical polymerization of acrylates.

**Figure 5:**
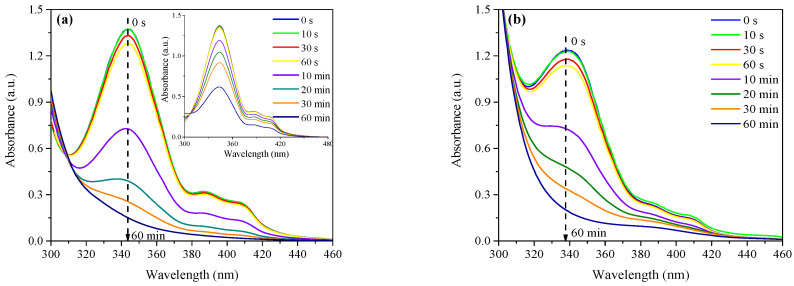
The steady state photolysis of (**a**) SQM1/I1 and (**b**) SQM1/I84 in *N,N*-dimethylformamide (DMF) upon the argon ion laser exposure (I_0_ = 50 mW × cm^−2^). The concentration of iodonium salt was 1 × 10^−3^ M. Inset: The photobleaching of squaraine dye (SQM1) as a result of laser irradiation.

The steady state photolysis experiments showed that the exposure of the dye solution to laser radiation results in a gradual bleaching of photosensitizer. It can be observed by the difference in light absorption. The longer exposure time of the sample causes the lower absorption intensity. For example, the changes in the intensity of absorption bands both of SQM1 dye solution, as well as the combination of squaraine dye SQM1 with iodonium salt, are presented in Figure 5.

As is clearly seen, the irradiation of squaraine dye solution results in a decrease in the intensity of absorption from about 1.5 to ca. 0.5 after 0 s and 60 min of irradiation, respectively. On the other hand, the presence of iodonium salt, I1, I81 and I84 leads to fast decrease of absorption intensity (even only 1 min of irradiation). The fastest photobleaching of squaraines was observed in the presence of (4-methoxyphenyl)-(4-nitrophenyl) iodonium *p*-toluenesulfonate (I81). The absorbance quenching processes are similar to that, when squaraine dye is combined with diphenyliodonium chloride (I1) or (3-bromophenyl)-(4-methoxyphenyl)iodonium *p*-toluenesulfonate (I84).

Therefore, the fastest interaction of SQ/Iod pair after light action is observed for the combination of squaraine dye with I81 salt. In other cases, the interaction dye-iodonium salt is similar. These differences may be observed in the photoinitiation efficiencies of novel radical photoinitiators.

### 3.2. Kinetic Studies of Photopolymerization Process

The influence of combinations of different photosensitizers in the form of squaraine dye with various diphenyliodonium salts on the kinetics parameters of the radical polymerization of trimethylolpropane triacrylate (TMPTA) was estimated. The photopolymerization experiments were conducted for different pairs of photosensitizer/co-initiator. The squaraine dyes, such as: 1,3-*bis*(benzothiazoleamino)squaraine (SQM1), 1,3-*bis*(6-bromobenzothiazoleamino)squaraine (SQM2) and 1,3-*bis*(6-methylbenzothiazoleamino)squaraine (SQM3) were used as UV-Vis light absorbers. Three diphenyliodonium salts: diphenyliodonium chloride (I1), (4-methoxyphenyl)-(4-nitrophenyl) iodonium *p*-toluenesulfonate (I81) and (3-bromophenyl)-(4-methoxyphenyl)iodonium *p*-toluenesulfonate (I84) played the role of co-initiators. The photopolymerization experiments were carried out for different initiator concentrations.

**Figure 6:**
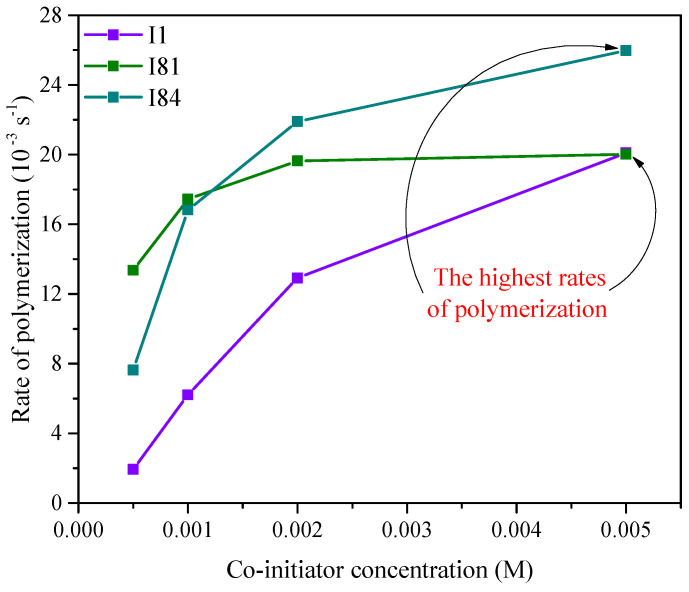
The effect of concentration of photoinitiator on the rate of the radical polymerization of TMPTA. The concentration of 1,3-*bis*(6-methylbenzothiazoleamino)squaraine (SQM3) was the same as for appropriate co-initiators and was marked on the figure.

In order to find the most optimal concentration of the photoinitiator in polymerizable mixture, that gives the highest kinetic parameters of radical polymerization of TMPTA, the kinetic studies for systems containing of 5 × 10^−4^ M, 1 × 10^−3^ M, 2 × 10^−3^ M and 5 × 10^−3^ M of SQ/Iod pair were carried out. The influence of initiator concentration on the rate of polymerization reaction of acrylate monomer was illustrated in Figure 6. The experiments were performed for all combinations of squaraine dye and diphenyliodonium salt. For example, the kinetic results of polymerization reaction for different concentration of SQM3/Iod pairs were presented in Table 2.

As shown in Figure 6, the concentration both of the sensitizer and the co-initiator, had an important impact on the kinetics of radical polymerization of triacrylate (TMPTA). The rate of polymerization increases as the initiator concentration in the polymerization mixture changed from 5 × 10^−4^ M to 5 × 10^−3^ M. The rate-initiator concentration curves for SQM3/I1 and SQM3/I84 are similar. The highest R_p_ values were observed for the highest concentration of photoinitiator, i.e., 5 × 10^−3^ M. It should be noted that concentrations of initiators of 2 × 10^−3^ M and 5 × 10^−3^ M give similar and the highest rates of polymerization, at about 20 × 10^−3^ s^−1^.

On the basis of the data summarized in Table 2, it can be concluded, that the highest final monomer conversions are observed for the photoinitiating systems comprised of 5 × 10^−3^ M of photoinitiator. The values of degree of double bond monomer conversion ranging from about 10% for SQM3/I1 photoinitiating system (concentration of initiator: 5 × 10^−4^ M) to above 35% for SQM3/I81 combination (concentration of initiator: 5 × 10^−3^ M). Similar results were obtained for combinations of SQM1 and SQM2 dyes with mentioned iodonium salts. It needs to be highlighted, that using of initiator concentration of 2 × 10^−3^ M or 5 × 10^−3^ M are the best options due to the highest kinetic parameters of radical polymerization of TMPTA.

**Figure 7:**
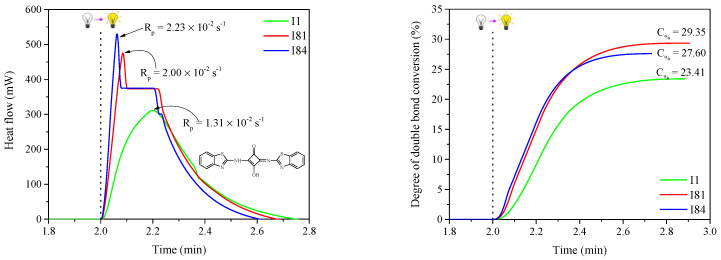
The kinetic curves registered during radical polymerization of TMPTA initiated by two-component photoinitiating systems composed of 1,3-*bis*(benzothiazoleamino)squaraine (SQM1) in the presence of various co-initiators marked on the figure. The concentration of photoinitiator was 5 × 10^−3^ M. The light intensity was 30 mW × cm^−2^.

In order to explain the influence of the type of co-initiator on the kinetics of the photopolymerization process, the efficiency of photoinitiating systems consisting of 1,3-*bis*(benzothiazoleamino)squaraine (SQM1) and various types of diphenyliodonium salts (I1, I81 was I84) was compared, what is shown in Figure 7.

From the kinetics data presented in Figure 7 and summarized in Table 3, it can be concluded that the structure of the co-initiator has a significant effect the kinetics of the polymerization reaction. The highest rates of polymerization and final monomer conversion were obtained from pairs composed of squaraine dye and (3-bromophenyl)-(4-methoxyphenyl)iodonium *p*-toluenesulfonate (I84). The exothermal effect for these photoinitiating systems was the highest and ranged from 300 mW to above 600 mW. The rates of polymerization achieved values about 2.20 × 10^−2^ s^−1^ to 2.60 × 10^−2^ s^−1^ and the final conversion ranged from 28% to 34%.

The photoinitiation reaction of radical polymerization of acrylate monomer (TMPTA) is very fast. It should be noted that the light action of the tested sample causes an immediate reaction with the release of a large amount of heat. The photocuring of polymerizable mixture takes only few seconds. As was shown in Table 3, The maximum of released heat during exothermal reaction was in the range from 12 s to even ca. 3 s for the most effective photoinitiator.

**Figure 8:**
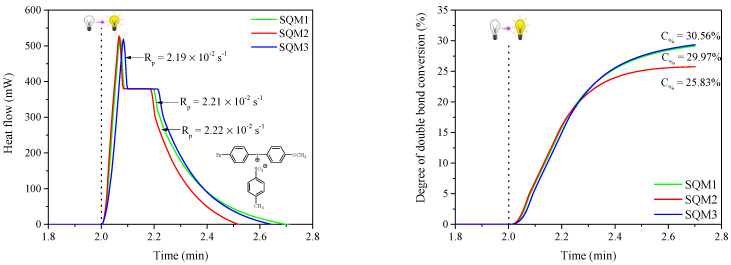
The kinetic curves registered during radical polymerization of TMPTA initiated by various two-component photoinitiating systems composed of various SQ dye and (3-bromophenyl)-(4-methoxyphenyl)iodonium *p*-toluenesulfonate (I84). The concentration of photoinitiator was 2 × 10^−3^ M. The light intensity was 30 mW × cm^−2^.

In this paper, we studied also the influence of sensitizer structure on the polymerization process, what was presented in Figure 8.

Apart from the initiator concentration and the structure of the co-initiator, the type of photosensitizer used in photopolymerization experiments also has a significant impact. The highest values of the kinetic parameters of reaction were obtained for photoinitiating system comprised of 1,3-*bis*(6-methylbenzothiazoleamino)squaraine (SQM3). Similar results were obtained for photoinitiators and consisted of SQM1 and SQM2 dyes. The final monomer conversion for photoinitiating systems containing SQM3 squaraine oscillated at about 35%. On the other hand, the combination of SQM1 or SQM2 squaraine dyes with all iodonium salts gives the degree of double-bond conversion in the range from 20% to 30%. It should be also noted that the highest values of photoinitiation indexes were obtained for bimolecular photoinitiators composed of squaraine dye and (3-bromophenyl)-(4-methoxyphenyl)iodonium *p*-toluenesulfonate (I84). In this case, this parameter is about 10 × 10^−3^ s^−2^.

On the basis of obtained kinetic results, one can conclude, that proposed bimolecular photoinitiators are very efficient and high-speed photoinitiating systems, which initiate the radical polymerization of acrylates with promising final monomer conversions. The further modification of system compositions may improve the kinetic parameters of radical polymerization of acrylate monomers. In the next papers, we focused on the combination of these 2-aminobenzothiazole derivatives with other co-initiator to improve the photoinitiating ability of these systems.

## 4. Conclusions

The proposed two-component photoinitiating systems consisting of newly synthesized 2-aminobenzothiazole derivatives and different diphenyliodonium salts may be used as ultraviolet-visible light active photoinitiators for the radical polymerization of trimethylolpropane triacrylate (TMPTA). The photoinitiation efficiency of novel photoinitiators depends on the sensitizer and co-initiator structures and their concentration in polymerizable composition. The highest values of kinetics parameters of radical polymerization of TMPTA were obtained for combination of squaraine derivatives (SQM1-SQM3) with (3-bromophenyl)-(4-methoxyphenyl)iodonium *p*-toluenesulfonate (I84). The rates of polymerization oscillates at about 2 × 10^−2^ s^−1^ and the total monomer conversion ranged from 20% to above 35%. The proposed photoinitiating systems may be used as effective high-speed initiators for radical polymerization of acrylate monomers under sensitive light conditions.

## Figures and Tables

**Table 1:** The spectroscopic properties of investigated photosensitizers.

Compound	λ_ab (max)_ (nm)	ε_(max)_ (dm^3^ × mol^−1^ × cm^−1^)	λ_fl (max)_ (nm)	Δν_St_ (cm^−1^)	Φ_fl_ (10^−2^)
SQM1	344	24,500	523	9949	1.64
SQM2	348	9700	538	10,148	1.17
SQM3	345	8300	418	5062	1.40

**Table 2:** The influence of photoinitiator concentration for kinetic parameters of radical polymerization of TMPTA initiated by squaraine dye (SQM3) with the combination of different iodonium salts: I1, I81 and I84.

Photoinitiator	Photoinitiator Concentration (M)							
	5 × 10^−4^		1 × 10^−3^		2 × 10^−3^		5 × 10^−3^	
	R_p_ (×10^−3^ s^−1^)	C_%_ (%)	R_p_ (×10^−3^ s^−1^)	C_%_ (%)	R_p_ (×10^−3^ s^−1^)	C_%_ (%)	R_p_ (× 10^−3^ s^−1^)	C_%_ (%)
SQM3/I1	1.93	12.25	6.21	26.43	12.92	29.56	20.10	34.27
SQM3/I81	9.94	25.16	20.02	34.70	21.77	35.68	23.37	35.71
SQM3/I84	7.64	24.63	16.82	30.00	21.90	30.56	25.98	33.45

**Table 3:** The kinetics parameters of radical polymerization of TMPTA initiated by bimolecular photoinitiators composed of 2-aminobenzothiazole derivative (SQM1/SQM2/SQM3) and various radical sources (I1/I81/I84). The concentration of photoinitiator was 5 × 10^−3^ M.

Photoinitiator	Co-Initiator	Q_(max)_ (mW)	t_(max)_ (s)	R_p_ (×10^−2^ s^−1^)	I_p_ (×10^−3^ s^−2^)	C_%_ (%)
SQM1	I1	311	12.03	1.31	1.09	23.41
	I81	475	5.03	2.00	3.98	29.35
	I84	529	3.83	2.23	5.82	27.60
SQM2	I1	465	6.23	1.96	3.15	25.70
	I81	547	4.03	2.31	5.73	28.18
	I84	612	2.83	2.58	9.12	29.68
SQM3	I1	476	8.23	2.01	2.44	34.27
	I81	554	4.23	2.34	5.53	35.71
	I84	616	3.23	2.60	8.05	33.45

## Data Availability

Not applicable.

## Supplementary Materials

The following are available online at https://www.mdpi.com/article/10.3390/ma14247814/s1, Figure S1: 1H NMR spectrum of SQM1 regis-tered in DMSO-d6, Figure S2: 13C NMR spectrum of SQM1 registered in DMSO-d6, Figure S3: 1H NMR spec-trum of SQM2 registered in DMSO-d6, Figure S4: 13C NMR spectrum of SQM2 registered in DMSO-d6, Figure S5: 1H NMR spectrum of SQM3 registered in DMSO-d6, Figure S6: 13C NMR spectrum of SQM3 registered in DMSO-d6.
