# Supplementary material for: High-Performance UV-Vis Light Induces Radical Photopolymerization Using Novel 2-Aminobenzothiazole-Based Photosensitizers
Source: Materials (Basel). 2021 Dec 17;14(24):7814. doi: 10.3390/ma14247814 (PMC8705399; doi:10.3390/ma14247814)
Supplement: Supplementary file 1 [file materials-14-07814-s001.zip › materials-1495127-supplementary.pdf]

materials

High-Performance UV-Vis Light Induces Radical Photopolymerization Using Novel 2-Aminobenzothiazole-Based Photosensitizers

Alicja Balcerak [1,*], Janina Kabatc [1], Zbigniew Czech [2,*], Małgorzata Nowak [2] and Karolina Mozelewska [2]

[1] Department of Organic Chemistry, Faculty of Chemical Technology and Engineering, Bydgoszcz University of Science and Technology, Seminaryjna 3, 85-326 Bydgoszcz, Poland; nina@pbs.edu.pl
[2] International Laboratory of Adhesives and Self-Adhesive Materials, Department of Chemical Organic Technology and Polymeric Materials, Faculty of Chemical Technology and Engineering, West Pomeranian University of Technology in Szczecin, Pułaskiego 10, 70-322 Szczecin, Poland; nowak.malgorzata@zut.edu.pl (M.N.); karolina_mozelewska@zut.edu.pl (K.M.)
* Correspondence: alicja.balcerak@pbs.edu.pl (A.B.); psa_czech@wp.pl (Z.C.)

Table of contents

	Page
^{1}H and ^{13}C NMR spectra of SQM1-SQM3	2 - 4

Citation: Balcerak, A.; Kabatc, J.; Czech, Z.; Nowak, M.; Mozelewska, K. High-Performance UV-Vis Light Induces Radical Photopolymerization Using Novel 2-Aminobenzothiazole-Based Photosensitizers. *Materials* **2021**, *14*, 7814. https://doi.org/10.3390/ma14247814

Academic Editor: Gerard Lligadas

Received: 19 November 2021
Accepted: 13 December 2021
Published: 17 December 2021

Publisher's Note: MDPI stays neutral with regard to jurisdictional claims in published maps and institutional affiliations.

Copyright: © 2021 by the authors. Submitted for possible open access publication under the terms and conditions of the Creative Commons Attribution (CC BY) license (http://creativecommons.org/licenses/by/4.0/).

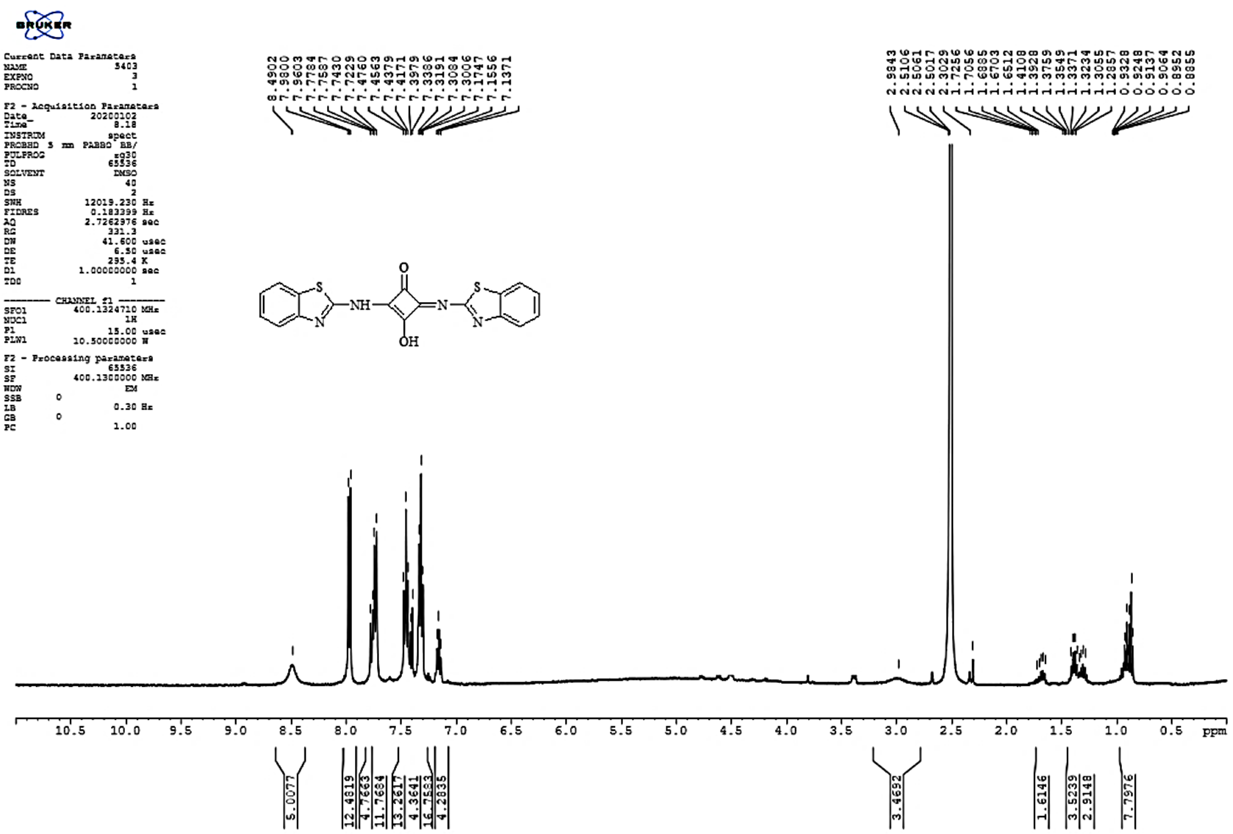

Figure S1. ^{1}H NMR spectrum of SQM1 registered in DMSO-d_6.

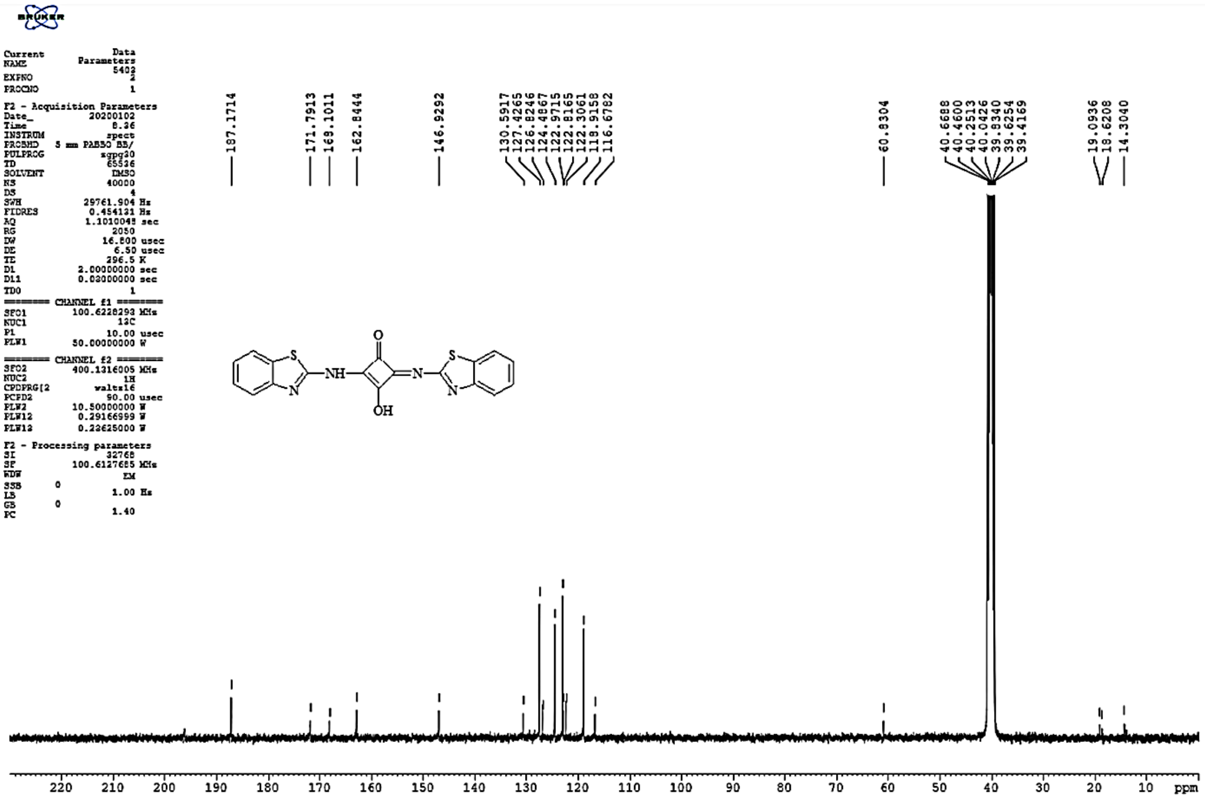

Figure S2. ^{13}C NMR spectrum of SQM1 registered in DMSO-d_6.

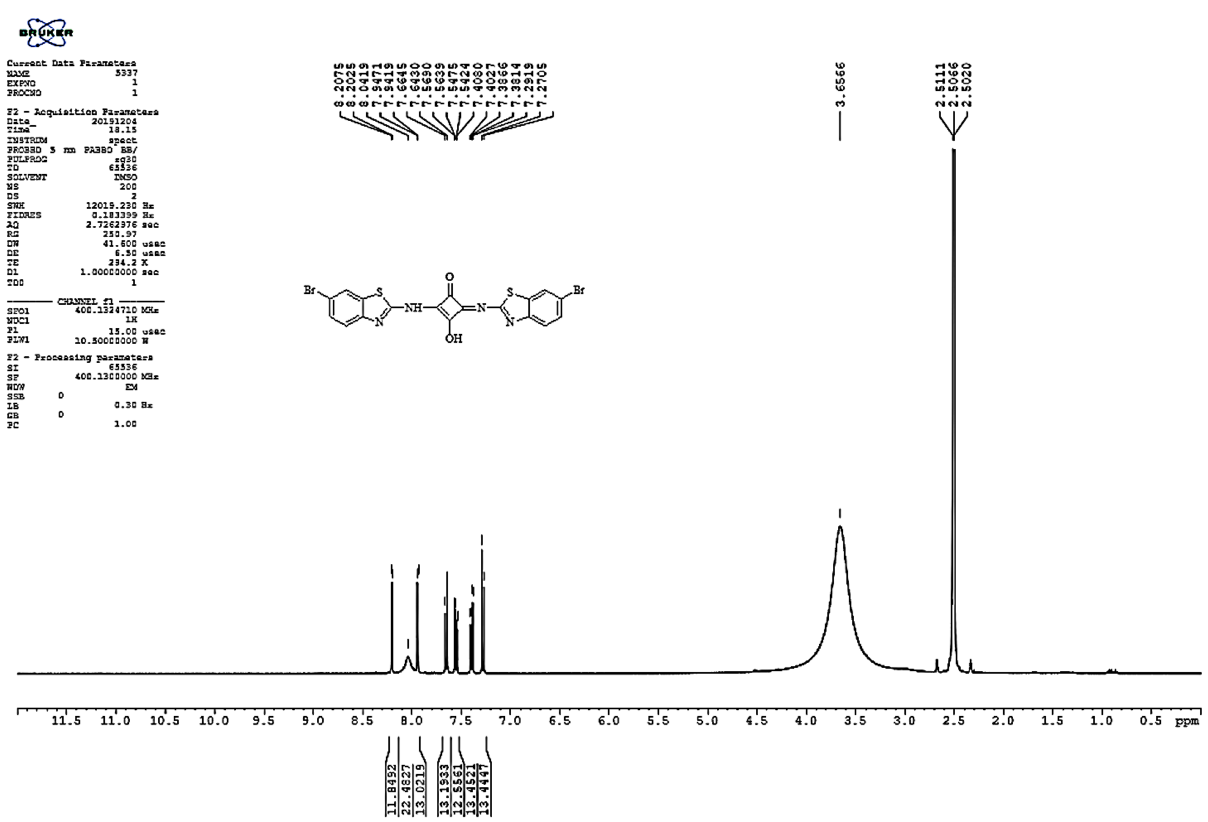

Figure S3. ^{1}H NMR spectrum of SQM2 registered in DMSO-d_6.

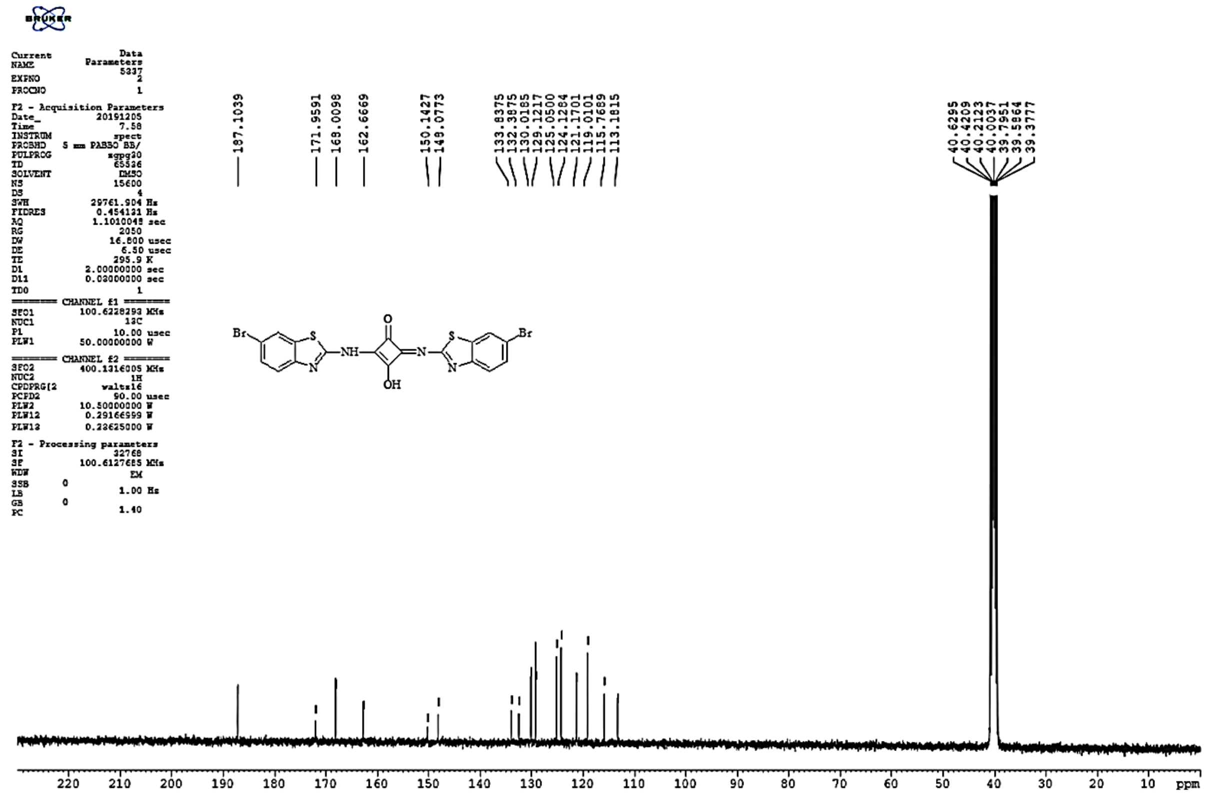

Figure S4. ^{13}C NMR spectrum of SQM2 registered in DMSO-d_6.

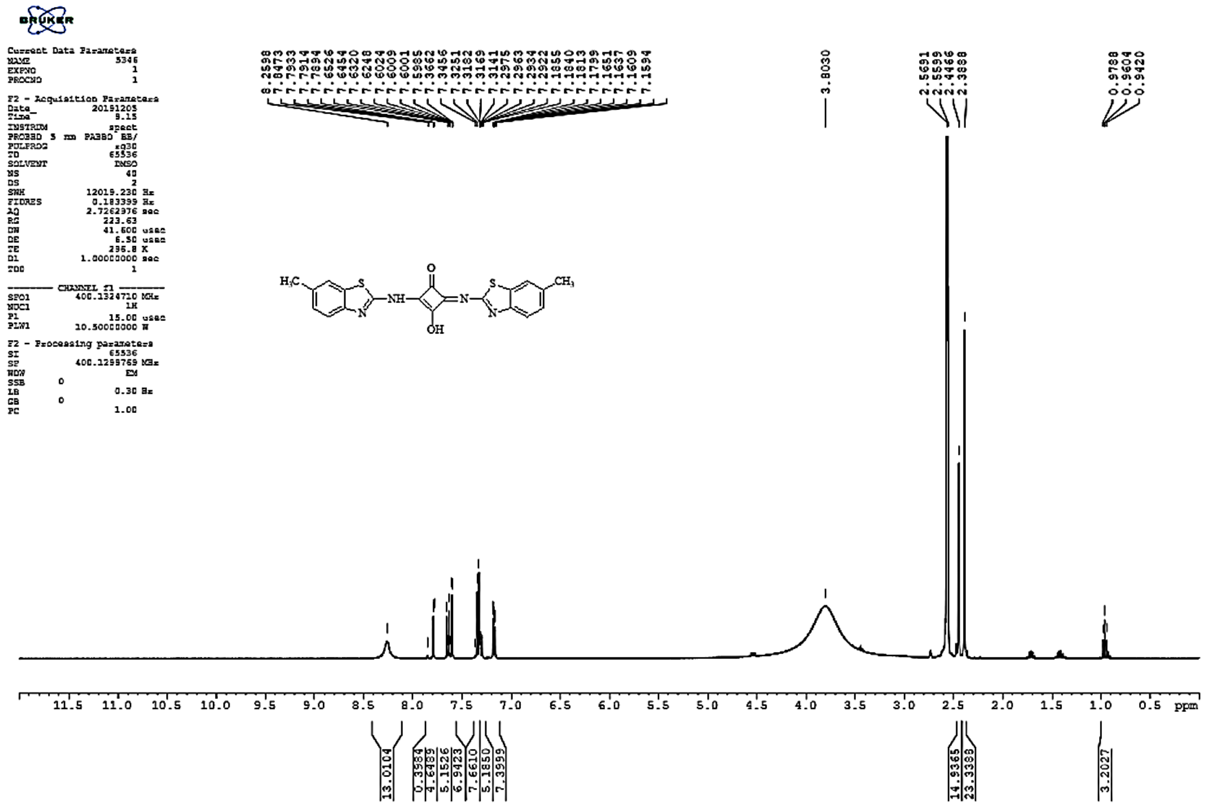

Figure S5. ^{1}H NMR spectrum of SQM3 registered in DMSO-d_6.

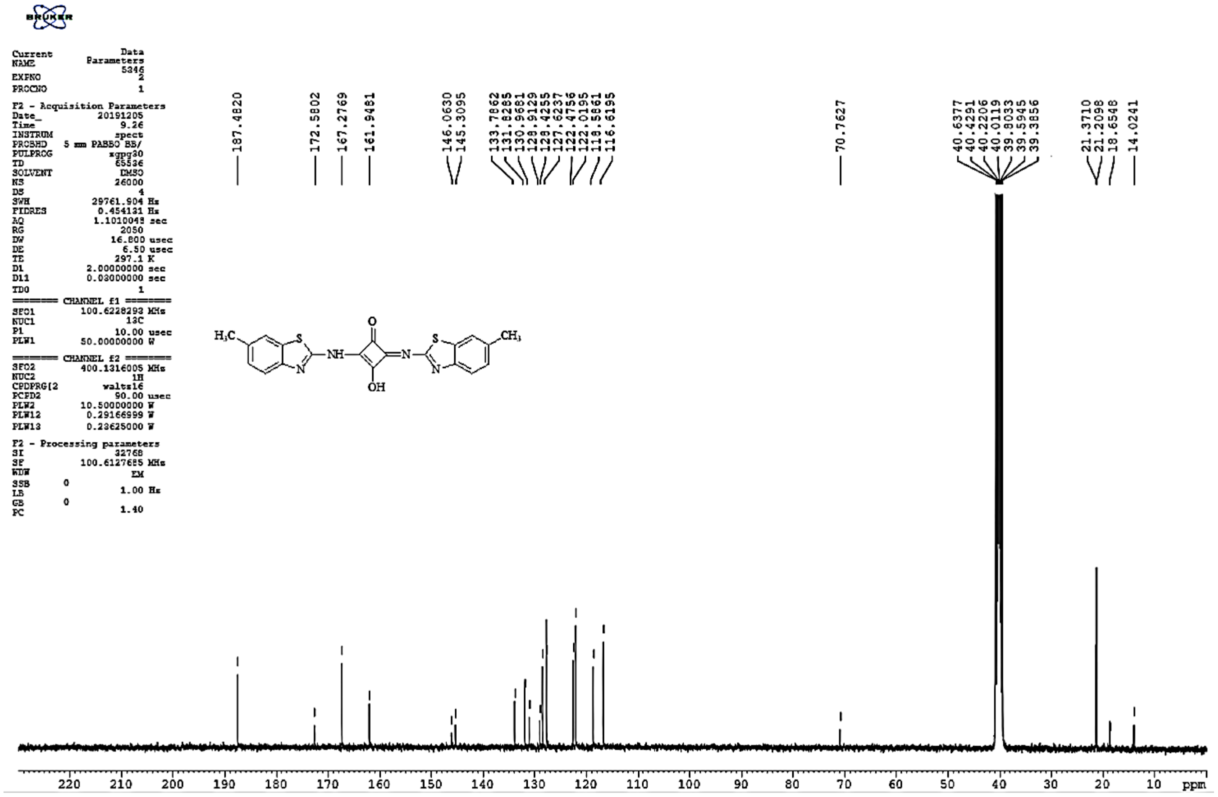

Figure S6. ^{13}C NMR spectrum of SQM3 registered in DMSO-d_6.
